# Pathogenesis of IgA Nephropathy: Current Understanding and Implications for Development of Disease-Specific Treatment

**DOI:** 10.3390/jcm10194501

**Published:** 2021-09-29

**Authors:** Barbora Knoppova, Colin Reily, R. Glenn King, Bruce A. Julian, Jan Novak, Todd J. Green

**Affiliations:** 1 Department of Microbiology, University of Alabama at Birmingham, Birmingham, AL 35294, USA; barcak@uab.edu (B.K.); rgking@uab.edu (R.G.K.); bjulian@uabmc.edu (B.A.J.); 2 Department of Medicine, University of Alabama at Birmingham, Birmingham, AL 35294, USA; creily@uab.edu

**Keywords:** IgA nephropathy, *O*-glycosylation, IgA1, autoantibody, immune complex

## Abstract

IgA nephropathy, initially described in 1968 as a kidney disease with glomerular “intercapillary deposits of IgA-IgG”, has no disease-specific treatment and is a common cause of kidney failure. Clinical observations and laboratory analyses suggest that IgA nephropathy is an autoimmune disease wherein the kidneys are damaged as innocent bystanders due to deposition of IgA1-IgG immune complexes from the circulation. A multi-hit hypothesis for the pathogenesis of IgA nephropathy describes four sequential steps in disease development. Specifically, patients with IgA nephropathy have elevated circulating levels of IgA1 with some *O*-glycans deficient in galactose (galactose-deficient IgA1) and these IgA1 glycoforms are recognized as autoantigens by unique IgG autoantibodies, resulting in formation of circulating immune complexes, some of which deposit in glomeruli and activate mesangial cells to induce kidney injury. This proposed mechanism is supported by observations that (i) glomerular immunodeposits in patients with IgA nephropathy are enriched for galactose-deficient IgA1 glycoforms and the corresponding IgG autoantibodies; (ii) circulatory levels of galactose-deficient IgA1 and IgG autoantibodies predict disease progression; and (iii) pathogenic potential of galactose-deficient IgA1 and IgG autoantibodies was demonstrated in vivo. Thus, a better understanding of the structure–function of these immunoglobulins as autoantibodies and autoantigens will enable development of disease-specific treatments.

## 1. Introduction

IgA nephropathy (IgAN) is the most common form of primary glomerulonephritis in many countries [1]. It was initially described in 1968 by Drs. Jean Berger and Nicole Hinglais as a kidney disease with glomerular “intercapillary deposits of IgA-IgG” [2]. Five decades later, the diagnosis still requires examination of kidney tissue. Routine immunofluorescence microscopy reveals IgA as the predominant or co-dominant immunoglobulin in the glomerular immune deposits. This IgA has distinctive characteristics: it is restricted to the IgA1 subclass [3] and has less galactose in its *O*-glycans than does circulating IgA1 in healthy persons (galactose-deficient IgA1; Gd-IgA1) [4,5]. The immune proteins in the glomeruli of patients with IgAN generally include complement C3; IgG, IgM, or both, are often present [6,7]. Light microscopy typically shows glomerular injury as mesangial hypercellularity and increased mesangial matrix [8].

IgAN may affect individuals of nearly all ages, although the diagnosis is rare in children younger than five years of age. The incidence of IgAN peaks in the second and third decades of life [1,7]. In children and adolescents, painless visible hematuria, often concurrent with an infection of the upper respiratory or gastrointestinal tract, frequently heralds the onset of clinical disease. This manifestation may also accompany intense physical activity. Most patients with macroscopic (visible) hematuria have additional episodes over several years [9]. Visible hematuria due to IgAN rarely begins after age 40 years. For patients in their 30s and 40s, microscopic hematuria, with or without, proteinuria may be discovered at the time of routine health screenings. The magnitude of proteinuria varies widely between patients, although proteinuria without microscopic hematuria is uncommon. IgAN is a common cause of chronic kidney disease, particularly for patients with proteinuria persistently more than 1 g/day [10,11,12]. There is currently no disease-specific therapy and 30–40% of patients progress to kidney failure that reduces life expectancy by about 10 years [13].

The incidence of IgAN varies substantially between ethnic/racial groups, being highest in East Asians; the disease accounts for about 40% of all native-kidney biopsies in Japan, 25% in Europe, 12% in the United States, and less than 5% in central Africa [14]. Some of this variability can be explained by differences in health screening policies and biopsy practices between these regions, but genetic factors also likely contribute [15]. The incidence in the United States has been estimated at 1 per 100,000 person-years [16]. Distribution between the sexes varies by region for reasons not yet defined; the male:female ratio is 2–3:1 in North America [16,17,18] and Europe [19], but about 1:1 in East Asia [20]. The frequency of the disease may be underestimated. Autopsy studies found IgA glomerular deposits accompanied by glomerular pathology in 1.3% of victims of trauma in Finland [21] and 4% of victims in Singapore [22]. A Japanese study showed that 16% of kidney allografts (from living and deceased donors) had glomerular IgA deposits in biopsy specimens at the time of engraftment of which 10% exhibited histological features typical of IgAN [23]. A recent study confirmed these findings, with 13% of kidneys donated for transplantation exhibiting asymptomatic IgA deposition [24].

**Figure 1:**
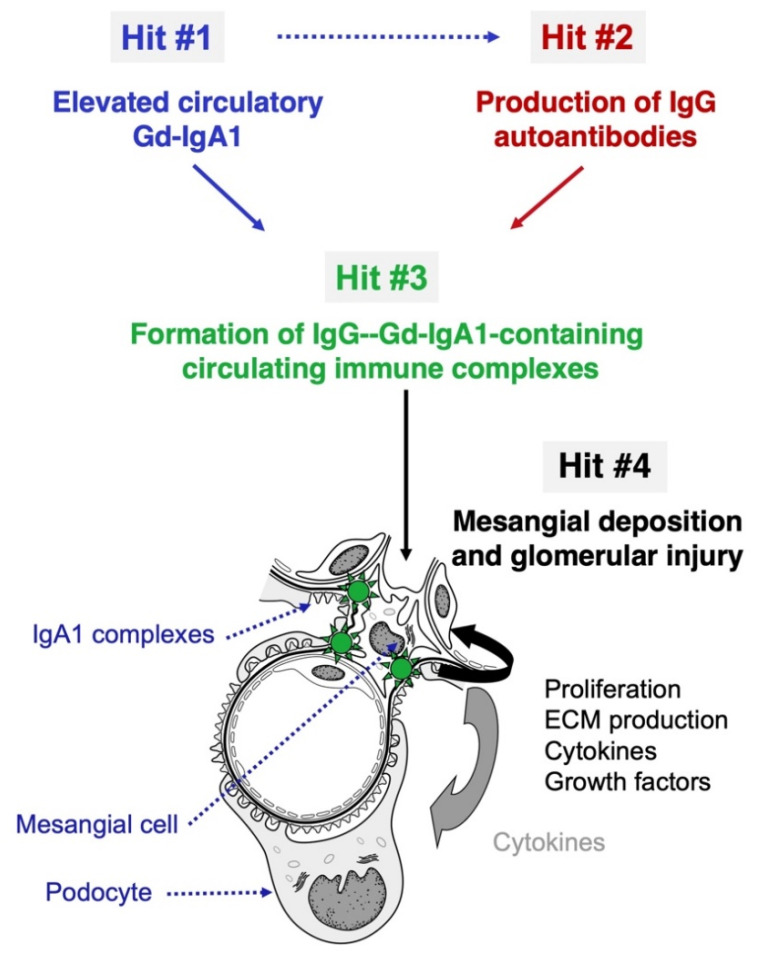
Model of pathogenesis of IgA nephropathy. IgA nephropathy (IgAN) is an autoimmune disease with a genetically and environmentally co-determined multi-hit process [31]. IgA1 with some *O*-glycans deficient in galactose (galactose-deficient IgA1; Gd-IgA1), in levels often elevated in the circulation of patients with IgAN (Hit #1), is recognized by IgG autoantibodies specific for Gd-IgA1 (Hit #2), and form pathogenic immune complexes, with other serum proteins being added (e.g., complement) (Hit #3). Blood levels of the autoantigen (Gd-IgA1) and the corresponding IgG autoantibodies correlate in IgAN patients, suggesting that elevated circulating levels of Gd-IgA1 are associated with the production of IgG autoantibodies specific for Gd-IgA1 (dashed arrow). Some of the immune complexes formed in the circulation deposit in the kidneys, activate mesangial cells, and induce glomerular injury (Hit #4). Figure modified with permission [44].

In IgAN, kidneys are injured innocently, as indicated by two key findings in kidney transplantation. IgAN often develops in allografts [25,26,27]; in contrast, IgA deposits disappear from allografts from donors with subclinical IgAN shortly after engraftment into non-IgAN recipients [28]. These observations suggest that the glomerular IgA is deposited from the circulation. While serum Gd-IgA1 levels are elevated in most patients with IgAN, such levels are not sufficient to induce the disease. Many first-degree relatives of patients with IgAN have comparably high levels for years without exhibiting any clinical feature of kidney disease [29]. Most of the circulating Gd-IgA1 is within immune complexes bound by IgG that recognizes galactose-deficient hinge-region (HR) *O*-glycans on the IgA1 heavy chain [30]. We have postulated that IgAN is an autoimmune disease with a multi-hit mechanism [31] (Figure 1): Gd-IgA1 is produced in greater quantities in IgAN patients compared with that in healthy individuals whereby circulating Gd-IgA1 levels are elevated (hit #1). These Gd-IgA1 molecules are recognized by IgG autoantibodies (hit #2), leading to formation of immune complexes in the blood (hit #3). Some of these circulating immune complexes accumulate in the glomerular mesangium and activate resident mesangial cells to induce kidney injury (hit #4). This proposed sequence is in agreement with the observations that serum levels of Gd-IgA1 (autoantigen) and the corresponding autoantibodies each correlate with disease severity and progression [30,32,33,34]. Furthermore, the IgG co-deposits in glomeruli are of the IgG1 and IgG3 subclasses [35] as are the IgG autoantibodies in the circulation [30]. IgG is the main autoantibody isotype; the levels of serum IgG autoantibodies in patients with IgAN correlate with those of the autoantigen, Gd-IgA1 [36], and predict disease progression and disease recurrence after transplantation [32,34,37,38,39]. Other studies revealed that IgG glomerular deposits were associated with a worse long-term outcome [40,41,42]. These studies together indicate the major role of IgG autoantibodies in IgAN pathogenesis [43].

Routine immunofluorescence microscopy fails to reveal IgG in many kidney biopsies of patients with IgAN [41,45]. To address this apparent discrepancy with the proposed multi-hit mechanism of disease, we characterized the IgG extracted from glomeruli of remnant renal-biopsy specimens of patients with IgAN to test its antigenic specificity [46]. IgG was detected in glomerular immunodeposits of all IgAN patients, including those without IgG on routine immunofluorescence microscopy. Furthermore, this IgG, but not that in kidney biopsies from patients with membranous nephropathy and lupus nephritis, was enriched for autoantibodies specific for Gd-IgA1. Confocal microscopy using an IgG-specific nanobody confirmed that IgG was present in all IgAN biopsies, irrespective of whether IgG was detected by routine immunofluorescence microscopy. The IgA and IgG co-localized in the glomeruli, indicating that these immunoglobulins comprised immune complexes [46]. These findings strengthen the postulated multi-hit mechanism for the development of IgAN and the central role of IgG autoantibodies in the process, as confirmed recently in an experimental animal model [47].

Most cases of IgAN appear to be sporadic, although kindreds with familial IgAN are well described [48,49]. Genetically determined factors contribute to the pathogenesis of IgAN, even in patients with apparently sporadic disease [50,51,52,53,54]. Genome-wide association studies of predominantly European and East Asian cohorts have identified at least 21 risk variants for IgAN [55]. Some variants affect the enzymes controlling the glycosylation of IgA1 [56,57,58] while others alter innate immunity or modulate the activity of the complement system [54,59]. The genetic variants identified so far account for about 7% of the disease risk. Moreover, the cumulative number of the risk variants is strongly associated with age at disease onset, increasing progressively with younger age at diagnosis [50,51,52,53,54].

## 2. Galactose-Deficient IgA1 (Hit #1)

In humans and higher primates, IgA exists in two isoforms (also called subclasses), IgA1 and IgA2 (~84% and ~16% of total circulatory IgA, respectively). Both isoforms can be in the monomeric and polymeric forms, mIgA, and pIgA. pIgA has a joining chain (J chain), a ~17-kDa protein that forms disulfide bridges with Cys residues at the tail piece of the α heavy chain on the Fc region, to join two IgA monomers [60]. Most pIgA1 and pIgA2 is produced in the mucosal tissues, where pIgA molecules are moved by transcytosis onto the mucosal surfaces. For mucosal secretions, pIgA is produced by IgA-secreting plasma cells in mucosal tissues, such as those in gut-associated lymphoid tissues (GALT) [61,62]. pIgA is bound at the basolateral face of the intestinal epithelial cells by the pIg-receptor (pIgR), which is responsible for transcytosis of pIgA or pIgM. Once the pIgR-IgA complex reaches the apical membrane, pIgR is cleaved to produce the secretory component (SC), and the protein complex containing pIgA and SC is secreted onto the mucosal surfaces as secretory IgA [63,64].

**Figure 2:**
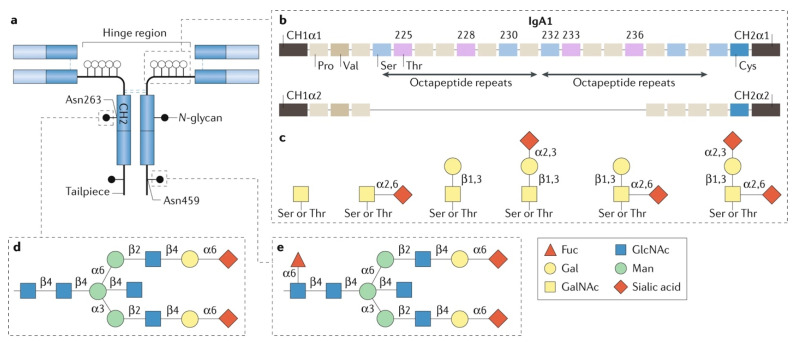
Structure and glycosylation of human IgA1. (**a**) Each heavy chain of IgA1 contains two *N*-glycans, one in the CH2 domain (Asn263) and the other in the tailpiece (Asn459). (**b**) The hinge-region (HR) of IgA1, unlike that of IgA2, contains nine Ser and Thr residues that are potential *O*-glycosylation sites. Only 3–6 of these residues are *O*-glycosylated and the most common IgA1 glycoforms have 4 or 5 *O*-glycans in the HR. (**c**) The *O*-glycan composition of normal circulatory IgA1 is variable but usually consists of a core 1 disaccharide structure with *N*-acetylgalactosamine (GalNAc) in β1,3-linkage with galactose (Gal); each of these monosaccharides can be sialylated. (**d**) The CH2 site of *N*-glycosylation contains digalactosylated biantennary glycans with or without a bisecting *N*-acetylglucosamine (GlcNAc), but it is not usually fucosylated. (**e**) The tailpiece *N*-glycosylation site contains fucosylated glycans. Figure reproduced with permission [43].

IgA1 has a unique HR between the first and second constant domains (CH1 and CH2) of the heavy chain, connecting the antigen-binding fragment (Fab, heavy-chain domains VH and CH1, and the entire light chain) with the Fc region. Each IgA1 HR contains 3–6 *O*-glycans, attached to some of the nine serine and threonine residues [65,66,67,68,69].The *O*-glycans of the HR of circulatory IgA1 are core 1 *O*-glycans. The biosynthesis is initiated by addition of an *N*-acetylgalactosamine (GalNAc) residue by a GalNAc-transferase (GalNAc-T), such as GalNAc-T2. This initial step may be followed by addition of galactose to GalNAc in a β1–3 glycosidic bond by the enzyme core 1 β1,3-galactosyltransferase (C1GalT1). Proper folding of the C1GalT1 protein is facilitated by its chaperone, C1GalT1C1 (COSMC), without which C1GalT1 protein integrity and, thus, function is compromised [70,71]. Additional modifications of GalNAc-galactose disaccharide can then occur on circulatory IgA1: GalNAc can then be sialylated via an α2–6 linkage and/or galactose sialylated via an α2–3 linkage (Figure 2) [72,73,74,75]. *O*-glycans of the HR consisting of GalNAc alone or sialylated GalNAc are galactose-deficient. The serum IgA1 in patients with IgAN has more galactose-deficient HR glycans (Gd-IgA1) than in healthy persons [76,77,78]. Furthermore, the glomerular IgA deposits are enriched for Gd-IgA1 glycoforms, most likely due to deposition of some of the circulating Gd-IgA1 [46]. The serum Gd-IgA1 level predicts progression to end-stage kidney disease (ESKD) [33]. For patients with ESKD who undergo transplantation, recurrent disease in the allograft is predicted by elevated Gd-IgA1 levels, supporting the hypothesis on the extra-renal origin of IgAN [24,37].

The mechanism(s) of Gd-IgA1 production in patients with IgAN is still under investigation. Gd-IgA1 in the circulation of patients with IgAN is predominantly polymeric [79] in contrast to the monomeric form for most of the circulatory IgA1. This fact raises questions about a potential mucosal origin of the Gd-IgA1-secreting cells and their localization (mucosal tissue vs. subsequent errant homing to bone marrow) [80,81]. Alteration in activity of several glycosylation enzymes has been associated with increased Gd-IgA1 production, including decreased expression and activity of C1GalT1 and increased expression and activity of ST6GalNAc2 [31,75,79,82]. The chaperone protein for C1GalT1, C1GalT1C1, is downregulated in B cells from IgAN patients compared to healthy controls and its activity inversely correlated with serum Gd-IgA1 levels [71]. Mechanistic studies found that some cytokines (e.g., leukemia inhibitory factor (LIF) and IL-6) increase Gd-IgA1 production in cultured IgA1-producing cell lines from IgAN patients but not in those from healthy controls. Specifically, supplementation of IL-6 and IL-4 in the media of cultured IgA1-producing cells from IgAN patients decreased expression and activity of C1GalT1 and increased expression of sialyltransferase ST6GalNAc2 [75]. IL-6-mediated activation increased and prolonged activation of the JAK-STAT pathway (detected as phospho-STAT3) was found in IgA1-secreting cells from IgAN patients compared to those from healthy controls [83]. Leukemia inhibitory factor (LIF) exhibits similar effects as IL-6 on IgA1-producing cells from IgAN patients, except that the signaling is mediated by STAT1. These cytokine effects underscore the role of aberrant signaling and cellular activation in Gd-IgA1 production in IgAN [84].

The synthesis of Gd-IgA1 is genetically co-determined. In patients with familial and sporadic IgAN, the serum level of Gd-IgA1 is a heritable trait; many blood relatives have higher levels than genetically distinct married-in relatives, but without any clinical manifestation of IgAN [29,85]. Genome-wide association studies (GWAS) of serum levels of Gd-IgA1 have identified single-nucleotide polymorphisms (SNPs) in the *C1GALT1* and *C1GALT1C1* loci that are related to the expression of specific genes. Reduced expression of *C1GALT1* and *C1GALT1C1* would be manifested by addition of less galactose to GalNAc of the IgA1 HR glycans. GWAS also revealed variants in several genes that influence the synthesis Gd-IgA1, including LIF [53,54].

Treatment of patients with IgAN has included several approaches that reduce circulating levels of Gd-IgA1. Tonsillectomy has been widely accepted in Japan [86,87,88,89,90]. Gd-IgA1 production by IgA1-secreting cells in tonsils is related to abnormalities in the expression of glycosyltransferase genes associated with core 1 *O*-glycosylation [91]. However, tonsillectomy has not improved kidney outcomes of IgAN patients in western Europeans, suggesting that the underlying mechanisms of Gd-IgA1 production may be distinct in different populations [92,93]. Although oral administration of corticosteroids may reduce Gd-IgA1 synthesis and, consequently, circulating levels of Gd-IgA1-IgG immune complexes [91,94,95], such therapy is not standard of care for most patients due to significant toxicity [96]. In the recent NEFIGAN trial, oral administration of a slow-release corticosteroid (TRF-budesonide) designed to reach the mucosal surface of the ileum significantly reduced proteinuria, slowed decline in kidney clearance function, and lowered serum levels of Gd-IgA1 and Gd-IgA1-IgG immune complexes [97,98,99].

Catabolism of circulatory polymeric and monomeric IgA occurs in the liver and depends on the asialoglycoprotein receptor (ASGPR) on hepatocytes [100,101]. The binding to ASGPR is dependent on the glycosylation status of IgA, in line with the well-known specificity of this hepatocyte lectin for glycans [102,103,104,105,106]. Circulating IgA1-IgG complexes may not be cleared as quickly in IgAN patients as in healthy controls. Clearance of an IgA1-IgG complexes-mimicking probe was delayed in IgAN patients compared with healthy controls) [107]. However, clearance of another protein probe (asialo α1 acid glycoprotein) was equivalent in the two cohorts, indicating that the liver clearance function is not generally impaired in IgAN patients.

The origins of Gd-IgA1 production have not been defined, despite many studies designed to evaluate the mechanisms of the synthesis of the immunoglobulin. There persists substantial technical difficulty in evaluating whether mucosal tissues are the site of Gd-IgA1-producing cells that secrete the galactose-deficient pIgA1 that enters the circulation. Additionally, as not all IgA1-secreting cells produce Gd-IgA1, many questions remain about how cytokines may control differential regulation of glycosylation in Gd-IgA1-producing cells. Future innovative single-cell analytics that identify signaling and transcriptional mechanisms in IgA1-secreting cells that produce Gd-IgA1 should find potential targets for novel, effective disease-specific therapy.

## 3. Autoantibodies (Hit #2)

Autoantibodies in patients with IgAN recognize the aberrantly glycosylated HR of IgA1 [30,108]. The specificity of serum IgG autoantibodies was assessed using a panel of well-defined glycoforms of potential antigens. Serum IgG of patients with IgAN exhibited significantly higher binding to enzymatically desialylated and degalactosylated IgA1, Fab fragment of Gd-IgA1 with *O*-glycosylated HR, and albumin-linked HR glycopeptide with three GalNAc residues compared to enzymatically galactosylated or sialylated Gd-IgA1 and albumin-linked HR peptide without any glycan. These experiments confirmed the specificity of autoantibodies for IgA1 with its HR containing terminal GalNAc [30,109]. Serum Gd-IgA1-specific antibodies can be of IgG, IgM, or IgA isotype, and it is thought that at least some of them can be induced by microbiota with GalNAc-containing epitopes [110,111,112,113]. Follow-up studies with serum specimens from IgAN patients revealed that IgG is the predominant autoantibody isotype specific for Gd-IgA1 [36,114].

IgG autoantibodies specific for Gd-IgA1 in patients with IgAN have a distinctive sequence signature. IgG autoantibodies were cloned from immortalized IgG-producing cells derived from peripheral blood of several patients with IgAN using single-cell RT-PCR [30]. Sequence analysis of variable regions of the heavy chains (VH) of these IgG autoantibodies revealed serine in the junction of framework 3 and the complementarity determining region 3 (CDR3). Follow-up studies confirmed that serine residue in this region was important for binding to Gd-IgA1. When the recombinant IgG autoantibody from an IgAN patient was modified to replace serine with alanine in this position, the resultant IgG proteins exhibited reduced binding to Gd-IgA1. Conversely, IgG from a healthy control that weakly bound Gd-IgA1 had alanine in that amino-acid position; replacing it with serine significantly improved the binding, further confirming the importance of serine in this position for binding of IgG to Gd-IgA1 [30].

A follow-up study was performed to assess whether this single amino-acid alteration originates from a rare allele of a VH gene or somatic hypermutations, such as those occurring during antibody-maturation processes [115]. Germline genomic DNA from seven IgAN patients and six healthy controls was sequenced for the specific VH gene of each autoantibody. These genomic sequences were then compared with the sequences of the cloned VH regions of autoantibodies. Germline genomic VH genes of IgAN patients had nucleotide sequences encoding alanine or valine, but not serine. These findings identified several nucleotide mutations that resulted in a codon switch to serine. No such codon change was observed in sequences originating from healthy controls. These data indicate that serine in the framework 3-CDR3 region of these autoantibodies likely originates from somatic hypermutations that enhance the affinity for IgG autoantibodies in patients with IgAN [116].

It is not known what triggers the production of autoantibodies targeting IgA1 with terminal GalNAc residue(s) in the hinge-region. One hypothesis proposes that an infection by microorganisms carrying GalNAc on their outer surfaces elicits production of GalNAc-recognizing antibodies that cross-react with Gd-IgA1. Infection by Epstein–Barr virus, respiratory syncytial virus, herpes simplex virus, and streptococci may induce production of such antibodies [110,111,112,113,117]. Tn-antigen-specific antibodies (i.e., recognizing GalNAc-containing glycoconjugates) have been induced by ingestion or inhalation of live or killed *Escherichia coli* (O86) [118,119]. Upon infection of mucosal surfaces and gastrointestinal tract, susceptible individuals could react by producing hypermutated IgG against mucosal pathogens [120]. A recent study proposed an association between infection by *Streptococcus mutans* and severe outcome of IgAN [121]. Moreover, a study with *Bacteroidetes* bacteria found aberrant mucosal immune responses to tonsillar anaerobic microbiota and production of IgA specific for these bacteria may be involved in the pathophysiology of IgAN [122].

Regardless of the origin of Gd-IgA1-specific autoantibodies, their serum levels correlate with disease severity [30] and predict disease progression [32,34]. Therefore, measurement of serum levels of these autoantibodies may be a prognostic biomarker of IgAN. Furthermore, serum levels of Gd-IgA1-specific IgG autoantibodies correlate with the levels of serum Gd-IgA1 in IgAN patients [36], indicating potential utility of both biomarkers.

## 4. Other Types of Autoantibodies Targeting Aberrantly Glycosylated Proteins

Although the involvement of glycan-dependent Gd-IgA1-specific antibodies in IgAN is clear, the origin of these autoantibodies is currently unknown. Glycan-reactive antibodies are abundant in the circulation of healthy individuals and many specificities of these antibodies have been described. For example, antibodies against xenoantigens, such as α-galactose as well as allo- or auto-antigens, including blood groups which display potential self-reactivity, are all readily detectable [123]. Although high levels of self-reactive antibodies recognizing mature post-translational glycosylation are rare, antibodies that react with immature or truncated glycosylation precursors are abundant in humans. Many of these structures are also antigenic determinants of non-mammalian glycans, including commensal and pathogenic bacteria, which likely promote the production of most, if not all, natural glycan-reactive antibodies. Indeed, antibodies reactive with chitooligosaccharides, short polymers of *N*-acetylglucosamine (GlcNAc) residues that are structurally similar to the *N*-glycans, are among the most abundant glycan-specific antibodies in humans [124,125]. In rodents, the synthesis of these glycan-specific antibodies is driven by the commensal flora [124,125]. Whether Gd-IgA1-reactive antibodies are derived from naturally occurring precursors generated by microbial exposure or are the products of de novo responses against neoepitopes in Gd-IgA1 is unknown. These two possibilities are not mutually exclusive; a comparable scenario may be the antigenicity of tumor-associated antigen Tn-MUC1, i.e., MUC1 with terminal GalNAc residues (for review, see [126]).

Cellular transformation in cancer frequently alters glycosylation profiles. A similar process applies to the production of Gd-IgA1: the loss of function of the T-synthase (C1GalT1) or its molecular chaperone Cosmc (C1GalTC1) truncates the synthesis of the HR *O*-glycans after attachment of GalNAcα to serine or threonine [43]. The result is expression of tumor-associated antigen termed Tn antigen along with its sialylated form (sTn antigen). Similar to the glycan-reactive epitopes described above, the Tn and sTn antigens are among the epitopes covered by the natural antibody repertoire [127]. Moreover, like other glycan-specific antibodies, the anti-Tn reactivity in human serum antibodies is polyclonal in nature, exhibiting binding to an array of structurally similar glycans. In fact, human anti-Tn antibodies, including IgG and IgM isotypes, affinity-purified using terminal GalNAcα-containing matrix, demonstrate higher affinity for other glycan structures compared to terminal GalNAcα, including bacteria-derived products and cell-wall structures [128]. At least some of these antibodies clearly recognize tumor-associated Tn antigens [129]. MUC1 is a glycoprotein constitutively expressed by epithelial cells in many organs, including the lungs and gastrointestinal tract. Extensively glycosylated, 50% of the mass of MUC1 in normal healthy tissue is contributed by *O*-glycans [130]. Likely, because of its size, density of *O*-linked glycosylation, and high-expression levels in many tumors, aberrantly glycosylated MUC1 is an attractive tumor-associated antigen for immunotherapy. Considerable efforts have been devoted to harnessing antibodies toward aberrantly glycosylated MUC1 as cancer immunotherapy, through vaccination with glyco-MUC1 peptides or passive immunization [126].

As is the case with the other glycan epitopes described above, the fine-specificities of antibodies that recognize tumor-derived MUC1 are diverse, as are their antigenic targets. Immunization with MUC1 glycopeptides displaying aberrant glycan profiles generates antibodies that differ from the natural Tn-reactive antibodies [131]. Although direct binding to the Tn antigen has not been observed for most MUC1 antibodies generated in the efforts to develop anti-tumor therapeutics, Tn glycans appear to play a role in antigen binding. The affinity of many antibodies toward MUC1 peptides is increased in the presence of the Tn antigen, in some cases correlating with the number of Tn modifications on MUC1 peptides [132].

Several anti-MUC1 recombinant antibodies have been investigated as therapeutic reagents targeting breast, colon, and pancreatic cancers [133,134]. The mechanisms of action of the reagents vary, ranging from antibody-dependent cellular cytotoxicity to polarization of cell-associated MUC-1 promoting increased cell-mediated immunity [135,136]. For one such antibody, mAb-AR20.5 (BrevaRex), cellular and humoral immunity recognizing MUC1 were observed after its administration [137]. Initially, it was proposed that this mouse IgG1 antibody was activating the idiotypic network, resulting in the derivation of MUC1-reactive antibodies. It was subsequently determined, however, that the increase in anti-MUC1 serum antibody reactivity in patients receiving BrevaRex therapy was the result of the formation of immune complexes with BrevaRex and soluble MUC1 shed from tumors. These immune complexes promoted T cell-meditated and humoral immune activation directed toward the endogenous MUC1, resulting in elevated levels of antibody reactive with MUC1. Interestingly, a similar phenomenon has been observed for two other therapeutic antibodies targeting another mucin antigen (CA125) aberrantly glycosylated and shed from tumor cells [138,139]. Although there is much work to be done to determine the antigenic glycoforms of Gd-IgA1 contributing to IgAN and the autoantibodies that complex with Gd-IgA1 driving this disease, these examples offer a plausible mechanism by which the auto-immune responses to Gd-IgA1 could be initiated. Low affinity anti-Tn antibodies, which are universally present in human sera, may recognize Gd-IgA1 to form immune complexes, which, in turn, direct the activation of adaptive immunity, ultimately promoting epitope spreading and the generation of high-affinity pathogenic antibodies.

## 5. Circulating Immune Complexes (Hit #3)

Circulating immune complexes (CICs) in IgAN patients consist of IgG autoantibodies bound to polymeric IgA1 with galactose-deficient HR *O*-glycans [117,140,141]. Aggregates of Gd-IgA1 and fibronectin and extracellular matrix proteins have also been found in the circulation [142,143]. The latter study showed that besides IgG, CICs are composed of IgA, IgM, and complement C3 [144]. The biological activity of the CICs is determined by composition and size. The CICs containing high content of Gd-IgA1 induce proliferation of mesangial cells in culture, which was not observed for Gd-IgA1 alone or Gd-IgA1-lacking immune complexes [145]. Large-molecular-weight CICs (800–900 kDa) are biologically active. Cellular proliferation and overproduction of cytokines and components of extracellular matrix are observed in cultured primary mesangial cells stimulated with the large-molecular-weight CICs, whereas smaller complexes are inhibitory [145,146]. Experiments with in vitro produced immune complexes using Gd-IgA1 myeloma protein and anti-glycan IgG from cord blood of healthy women confirmed the stimulatory effects of IgA1-IgG immune complexes on mesangial cells [147].

**Figure 3:**
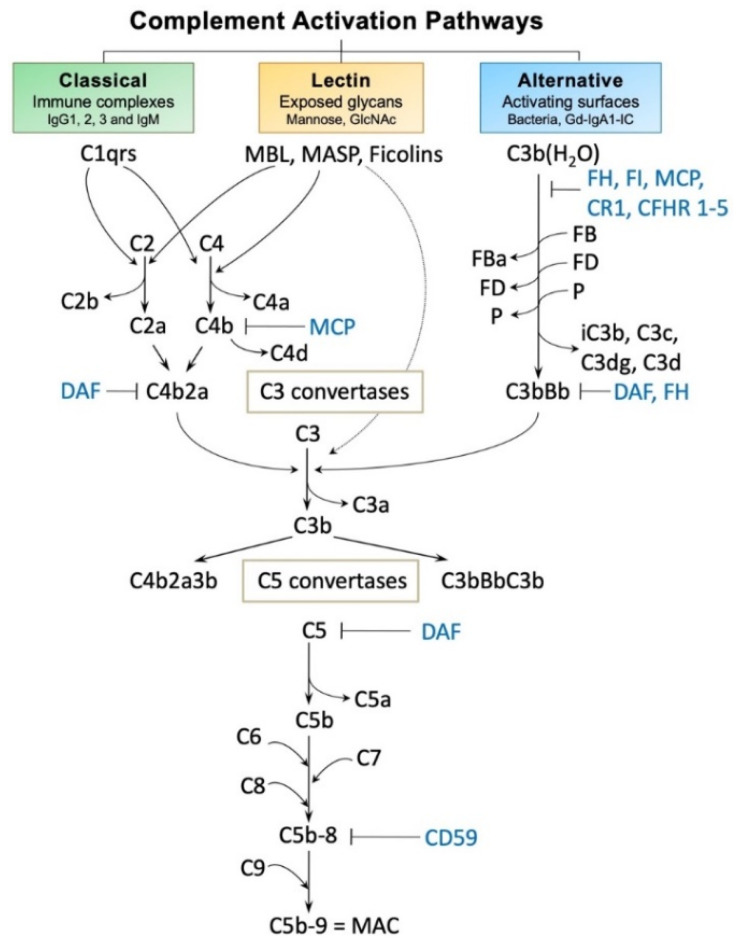
Complement activation pathways and selected regulatory proteins. The three pathways of complement activation, classical, lectin, and alternative, are initiated by interactions of complement proteins with distinct structures. The common activators for each pathway are described in the respective boxes. Complexes of antigen and antibody can activate the classical pathway. Mannan-binding lectin recognizes carbohydrate structures and, upon association with serine proteases (MASP, mannose-associated serine proteases), can activate the lectin pathway. Complement C3 that is covalently bound to microorganism surfaces as C3b initiates the cascade of the alternative pathway. Each pathway can ultimately generate an active C3 convertase, resulting in cleavage of C3 component into C3a and C3b fragments. C3b can interact with C4b2b or C3bBb to produce C5 convertase that cleaves C5 into C5a and C5b fragments. C5b binds to the cell membrane and serves as a platform for assembly of the membrane attack complex (MAC) [156,157]. The formation of MAC can be inhibited by membrane-bound CD59 that binds to C8 and/or C9. Selected regulatory proteins are shown in light blue. CR1, complement receptor 1; CFHR 1–5, complement factor H-related proteins 1–5; DAF, decay-accelerating factor; FB, factor B; FD, factor D; FI, factor I; Gd-IgA1, galactose-deficient IgA1; Gd-IgA1-IC, galactose-deficient IgA1-containing immune complexes; MCP, membrane cofactor protein; P, properdin. Figure modified with permission [151].

Complement component C3 [148] and its fragments (iC3b, C3c, and C3dg) are also in the Gd-IgA1-IgG immune complexes, confirming the biologic capability of these complexes to activate the alternative complement pathway [149]. In a study of 81 IgAN patients, C3 activation was demonstrated in 75% of the adult and 57% of the pediatric patients. Involvement of the classical complement pathway, assessed by C4 activation, was detected in plasma of 20% of the adult and 5% of the pediatric patients [150]. Analysis of kidney biopsy specimens reveals C3 in most cases, indicating involvement of alternative complement pathway, whereas C1 is usually absent. Complement activation pathways are described in Figure 3 (for review, see [151,152]). Recent proteomics analysis of CICs identified association of Gd-IgA1 with α1-microglobulin. Elevated blood levels of Gd-IgA1-α1-microglobulin correlated with hypertension, eGFR levels, and extent of scarring in the kidney biopsy sections [153].

The levels of CICs in IgAN patients correlate with clinical and histological activity such as microscopic hematuria, episodes of macroscopic hematuria, and severity of glomerular injury [154]. At the time of macroscopic hematuria, the blood level of immune complexes of IgA1-IgG may significantly increase, although IgA1-IgM complexes may also be present [155].

## 6. Deposition of Circulating Immune Complexes and Renal Injury (Hit #4)

As detailed above, IgAN is an autoimmune disease characterized by the glomerular deposition of immune complexes containing Gd-IgA1 and IgG autoantibodies specific for Gd-IgA1 [46,158,159]. These IgA1-containing glomerular immunodeposits usually contain also complement C3. As IgAN patients often have elevated levels of circulatory Gd-IgA1 and IgG autoantibodies, and the Gd-IgA1-IgG immune complexes also contain C3 and the same subclasses of IgG (IgG1 and 3), it is thought that the glomerular immunodeposits originate from the circulation [31].

In vitro experiments using cultured primary human mesangial cells [160] have shown that IgA1-containing immune complexes in the sera of patients with IgAN can activate mesangial cells [44,145,161,162,163,164,165,166,167,168,169,170,171]. In contrast, free (uncomplexed) Gd-IgA1 did not stimulate proliferation of cultured mesangial cells [31,145,146,147,164,171,172]. Moreover, immune complexes formed in vitro from Gd-IgA1 and IgG autoantibody mimicked the stimulatory effect of Gd-IgA1-IgG-containing immune complexes in sera of IgAN patients [147,159,173].

It is not fully understood what types of receptors on mesangial cells are engaged by the pathogenic complexes. Mesangial cells express several receptors that can bind IgA1: transferrin receptor (CD71) [166,174,175,176], integrin β1 [177], and cell surface galactosyltransferase (e.g., β1,4-galactosyltransferase) [178]. The transferrin receptor (CD71) binds IgA1, but not IgA2, and the IgA1 binding is inhibitable by transferrin [175]. CD71 binds polymeric, but not monomeric IgA1, and the binding is dependent on glycosylation, namely *O*-glycosylation [166]. CD71 is thought to participate in the binding of pathogenic IgA1-containing immune complexes by mesangial cells in IgAN [166]. The other two receptors, integrin β1 and cell surface β1,4-galactosyltransferase, can bind IgA1 molecules, although it is not clear whether either receptor is involved in the pathogenic cellular activation in IgAN.

IgA in a soluble or aggregated form can bind to FcαRI (CD89). Although mesangial cells do not express CD89, a complex formed from IgA1 and soluble CD89 can deposit in the kidneys, as shown in an experimental animal model [179]. It is not clear whether the same mechanism may operate in some IgAN patients. In general, IgA complexes can induce immunosuppressive or pro-inflammatory responses. Soluble forms of IgA, monomers and dimers, in the circulation have low affinity for FcαRI and bind only transiently, thus mediating inhibitory signaling under homeostatic conditions [180]. Similar inhibitory effects can be induced by peptidomimetics to reduce undesirable inflammatory responses, such as those triggered by abnormal IgA-containing immune complexes in IgA-mediated blistering skin diseases [181,182].

As noted above, glomerular immune deposits in patients with IgAN are enriched for Gd-IgA1 glycoforms. A recent study provided experimental in vivo evidence to underscore the pathogenic role of IgG autoantibodies specific for Gd-IgA1 [47]. Specifically, Gd-IgA1-IgG immune complexes injected into immunodeficient mice deposited in the glomerular mesangium, together with murine complement C3, and produced glomerular injury with histological features mimicking IgAN, such as mesangioproliferative changes. Injection of the individual components (IgA1 or IgG) did not have such effects.

Transcriptome profiling of kidney tissues from mice injected with Gd-IgA1-rIgG immune complexes revealed changes concordant with findings in kidney biopsy tissue from IgAN patients. Pathway-enrichment analysis showed that immune complexes formed by Gd-IgA1 and recombinant IgG (rIgG) dysregulated expression of genes in the MAPK signaling, phagosome, complement, and coagulation pathways, as well as in cell adhesion molecules, transcriptional misregulation, PPAR and Rap1 signaling, leukocyte migration, and osteocyte differentiation [47].

These experimental approaches demonstrate the key roles of aberrantly *O*-glycosylated IgA1 and the corresponding IgG autoantibodies in the formation of nephritogenic immune complexes. Future studies are needed to provide additional information about processes induced by glomerular deposition of Gd-IgA1-IgG immune complexes. It is hoped that this approach can serve as a basis for development of new tools for elucidating some aspects of pathogenesis of IgAN as well as for pre-clinical testing of future therapeutic approaches.

## 7. IgA Nephropathy—Disease-Specific Treatment Approaches

The multi-hit hypothesis for the pathogenesis of IgAN, as described above, provides an overview of the immune players, IgG autoantibodies and Gd-IgA1, and the pathogenic immune complexes that are at the core of the disease process. Despite having discovered these players, clarified the importance of the galactose-deficient HR *O*-glycans for formation of immune complexes, and developed the capacity to clone and characterize IgG autoantibodies, we still lack full understanding of how such disease-causing immune complexes are formed. Coincident with this, there is a lack of disease-specific treatment for IgAN and many patients progress to ESKD. Even the new ongoing clinical trials for IgAN target specific areas of the broader disease state [for review, see [183]]. Below, we describe conceptual premises for disease-specific treatment and methods that may allow investigators to get closer to that goal.

**Figure 4:**
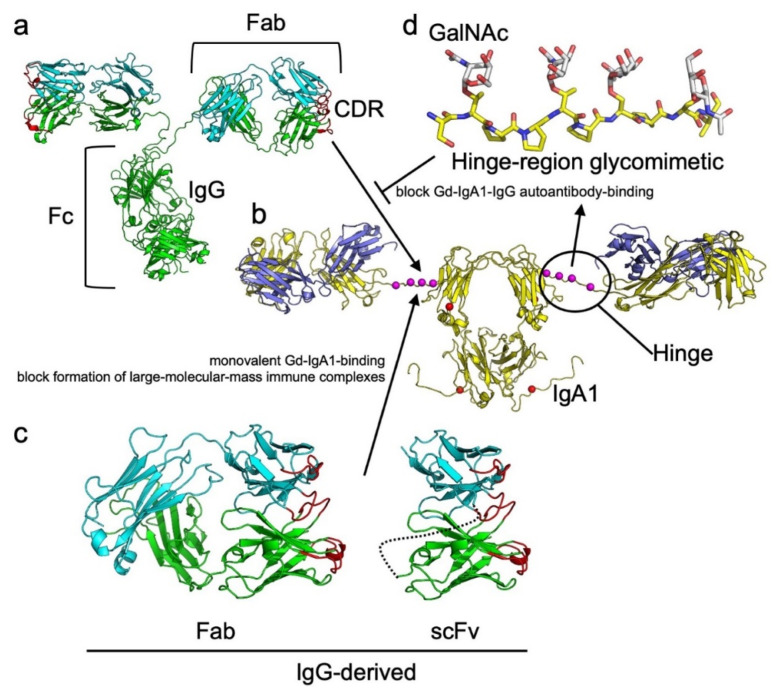
Structures of IgG and IgA1 with featured subdomain elements having theoretical utility to block formation of IgG-Gd-IgA1 complexes. In (**a**), an intact IgG (PDB ID: 1IGT, [184,185,186]) is shown in cartoon model. Heavy and light chains are green and cyan, respectively, with complementarity determining regions (CDRs) red. In (**b**), an intact IgA1 (based on PDB ID: 1IGA, [187,188,189,190]) is shown with heavy and light chains yellow and slate, respectively. Sites of *O*-linked (magenta) and *N*-linked glycans (red) are denoted by spheres. For simplicity, IgA1 is shown in monomeric rather than one of the possible polymeric forms. (**c**) Illustrates subdomain entities that could potentially be used to block IgG binding to Gd-IgA1. The Fab and scFv are both derived from the IgG and follow the same color patterns as in (**a**). The linker between VH and VL domains in the scFv is shown in dotted line. Due to the monovalent nature (single antibody-binding site) of these antibody fragments, each could bind to the Gd-IgA1, preventing IgG-binding and blocking formation of large-molecular-weight immune complexes. (**d**) A theoretical glycopeptide is shown in stick representation. The peptide, analogous to residues 224–233 of the IgA1 heavy chain, is shown with yellow carbon backbone. *O*-linked GalNAc (white carbon backbone) is shown linked at amino acids Thr-225, Thr-228, Ser-230, and Ser-232. This model could serve as a starting point for glycomimetic design for binding to IgG autoantibodies that target Gd-IgA1. Glycans were modeled with GLYCAM [191] and energy minimized with YASARA [192]. Images were made with PyMOL [193].

While effective in many cases, the current treatments for IgAN fail to address the key causation of the disease, formation of pathogenic immune complexes. In principle, methods to prevent or interfere with the process can be developed. From the perspective of the IgG autoantibodies, smaller versions of IgG that retain the capacity to bind Gd-IgA1, but are reduced to monovalent interactions, could be generated (Figure 4). Examples of such reagents include Fab antigen-binding fragments, single-chain variable fragments (scFv), or single-domain nanobody fragments [31]. These small monovalent reagents could potentially reduce formation of large pathogenic immune complexes by blocking binding of IgG autoantibodies to Gd-IgA1, thereby forming smaller complexes less prone to deposit in the kidneys. An alternative to this approach is to develop glycopeptides or smaller glycosylated proteins that are analogous to the HR of Gd-IgA1 or epitopes recognized by Gd-IgA1-specific IgG (Figure 4). These Gd-IgA1 glycomimetics could bind to IgG autoantibodies and prevent formation of complexes; however, design of minimalized versions of these Gd-IgA1 analogs is hampered by gaps in our knowledgebase regarding the definitive epitope(s) on Gd-IgA1 that are recognized by IgG autoantibodies.

One pathway to a better understanding how these disease-causing immune complexes are formed may be the use of structural biology, an area of science that aims to define the molecular structure of proteins, protein complexes, and other biological elements to better understand the correlation between structure and function. Techniques for this pursuit include x-ray crystallography (XRC), electron microscopy (EM), small-angle x-ray scattering (SAXS), nuclear magnetic resonance spectroscopy (NMR), and mass spectrometry (MS). These tools have been critical for defining the structures of antibodies [for a recent review of structural immunology, see [194]] and the glycan composition of IgA1 (see descriptions above). The first volume structure of an immunoglobulin, an IgG, was published in 1971 [195]. This low-resolution structure (6Å resolution) showed the three-dimensional (3D) organization of the IgG domains. In subsequent years, atomic structures of individual antibody domains and segments were published: the Fab (initially at 6Å resolution [196] and later as a high-resolution structure [197]), and the fragment-crystallizable region (Fc) [198]. Individual domains were then used to resolve the 3D coordinates of the first intact antibody in 1977 [199]. Further studies led to a small-molecule Fab structure; as a result, a basis for how antibodies bind to antigens was understood [197]. Since these early structures were published, three additional intact IgG structures have been published [184,185,186], which have collectively confirmed a common molecular structure. Although high-resolution structures of intact IgA1 are lacking, four low-resolution bead models derived from SAXS studies have been produced [187,188,189,190]. Moreover, 3D renderings of antibodies are illustrated in Figure 4. There are nearly 5200 antibody structures in the Research Collaboratory for Structural Bioinformatics Protein Data Bank [200], 85% of which were determined with XRC. These structural data provide valuable information about antibodies in general, but more specifically provide clues as to how they interact with antigens because most antibodies are in complexes with an antigen [201].

Despite this wealth of information, there is no structure of the glycosylated HR of IgA1 or structures of IgG or Fabs derived from patients associated with IgAN. In recent years, the field of cryo-EM has undergone what has been termed the “resolution revolution”, meaning that this technique has come of age, maturing to a high-resolution structural technique on par with XRC [202,203]. The advantage of cryo-EM is that the technique is amenable to proteins and protein complexes that are large and have some inherent flexibility, which can present issues with NMR and XRC techniques, respectively. Since 2020, structures of larger IgA1 segments and their complexes have been produced: Fc of secretory IgA1 in complex with J chain and the secretory component of pIgR (dimeric [204,205,206], tetrameric [204,206], and pentameric forms of IgA1 [204]), the dimeric form of IgA1 Fc in complex with pneumococcal adhesion protein, SpsA [206], and IgA1 in complex with the *Streptococcus pneumoniae* IgA1 protease [207]. These structures demonstrate feasibility to determine the structures of large IgA1-associated complexes using cryo-EM. The complex of IgA1 protease with IgA1 represents the first experimentally determined high-resolution partial structures of the IgA1 hinge-region, although the structure does not resolve *O*-glycans on the hinge-region.

As noted, a paramount goal in determining these structures is to provide a better understanding of function and, in part, to generate hypotheses on possible approaches to inhibit protein-protein associations. Still, the missing key elements in this pathway toward disease-specific therapy for IgAN include knowledge of structures of the complexes of IgG autoantibodies with Gd-IgA1autoantigen. Cryo-EM appears to be the likely avenue to acquire this information. To go a step further and better define the epitopes on Gd-IgA1, a panel of autoantibodies representing a comprehensive repertoire from patients with IgAN is needed. Each structure will presumably reveal a snapshot of a unique binding-interface. These data will provide a comprehensive topographical representation of the VH/VL surfaces involved in Gd-IgA1 recognition and differences in binding associated with IgG variability. Defining the same, overlapping, and/or distinct epitopes on the Gd-IgA1 will provide an opportunity to design strategies for disrupting the interaction of IgG and Gd-IgA1. It remains to be determined if small molecules can be designed that disrupt formation of pathogenic immune complexes, but discovery of the structures of each IgG autoantibody and Gd-IgA1 affords the opportunity to perform in silico screening of ligand libraries [208,209] in hopes of identifying, at least virtually, compounds that target critical areas of antibody-antibody binding. Compounds fitting this criterion could be validated by ELISA-based tests and further assessed in vitro (e.g., with cultured human mesangial cells) and in vivo in a small-animal model of IgAN [47]. It remains to be seen if these concepts for generating disease-specific treatments can be successfully implemented.

## 8. Conclusions

IgAN is characterized by glomerular immunodeposits enriched for Gd-IgA1 glycoforms and for IgG autoantibodies with specificity for the IgA1 with galactose-deficient *O*-glycans. These immunodeposits are thought to originate from Gd-IgA1-IgG complexes formed in the circulation. It was recently shown experimentally that human IgG autoantibodies bind to human Gd-IgA1 to form immune complexes that in a murine model induce pathogenic changes consistent with IgAN. It is hoped that a better understanding of the key players in IgAN, autoantibodies, and autoantigens, will enable development of disease-specific treatments.
